# A comparison of trueness and precision of 12 3D printers used in dentistry

**DOI:** 10.1038/s41405-022-00108-6

**Published:** 2022-05-26

**Authors:** Adam Nulty

**Affiliations:** 1 University of Leeds, Woodhouse Lane, Leeds, LS2 9JT UK; 2 International Digital Dental Academy, London, England

**Keywords:** Dental equipment, Restorative dentistry, Dental materials

## Abstract

**Introduction:**

Judging the dimensional accuracy of the resulting printed part requires comparison and conformity between the 3D printed model and its virtual counterpart. The resolution and accuracy of 3D model samples are determined by a wide array of factors depending on the technology used and related factors such as the print head/laser spot size/screen resolution, build orientation, materials, geometric features, and their topology.

**Aims:**

The aim of this manuscript is to present a literature review on 12 3D printers, namely the Ackuretta Sol, Anycubic Photon and Photon S, Asiga Max UV, Elegoo Mars, Envisiontec Vida HD, Envisiontec One, Envisiontec D4K Pro, Formlabs Form 2 and Form 3, Nextdent 5100, and Planmeca Creo, studying the accuracy of these printers that are of a wide variety of budgets.

**Design:**

The present study involves some of the recently released 3D printers that have not yet been studied for their accuracy. Since these new printers will replace current models that may have been included in the previous studies in the literature, it is important to study whether they are statistically more or less accurate and to discuss whether these results are clinically relevant. For the purposes of this study, the use of a standardised printable object was used to measure the accuracy of these recent 3D printers.

**Materials and methods:**

In total, 12 3D printers produced test blocks. All test blocks were printed using the same settings with 100 micron Z layer thickness and the print time set to standard where applicable. To measure the resulting blocks a digital measurement was taken using a Dentsply Sirona Ineos X5 lab scanner to measure the XYZ dimensions of each block produced on each printer using CloudCompare to measure the deviation compared to the Master STL. Each measurement was taken from the central axis of that dimension.

**Results:**

When grouped into homogenous subsets, the cheapest 3D printers in the group, namely the Anycubic printers and the Elegoo Mars, are statistically not dissimilar to the higher priced Asiga Max UV or even the mid-priced Formlabs printers in the X and Z dimensions. However, the Envisiontec One and D4K Pro, Ackuretta Sol and Asiga Max UV were statistically superior in terms of consistently accurate Y dimension. Although these printers use different technologies to print, no specific type of printer technology is more accurate than the others.

**Discussion:**

The null hypothesis was proved to be true, in that no significant differences were found among the various technologies of 3D printing regarding trueness and precision. The evolution of 3D printers that leads to budget printers being as statistically accurate, for at least two of the dimensions of data recorded, as expensive printers is remarkable. Whilst clear differences in the mean error between the printers were found, the performance of these printers is considered exceptional. Albeit, the Envision One, Envision D4K, Ackuretta Sol and Asiga Max UV printers performed the best with overall trueness under 35 μm.

**Conclusion:**

This study shows that the current range of 3D printers can produce clinically acceptable levels of accuracy. The present study also shows that there is no statistical difference in the results of budget printers and more expensive printers for the X and Z dimensions but this was not the case for the measurements in the Y dimension. This study confirms that all of the 3D printers can produce a reliable, reproducible model.

## Introduction

Judging the dimensional accuracy of the resulting printed part requires comparison and conformity between the 3D printed model and its virtual counterpart. The resolution and accuracy of 3D model samples are determined by a wide array of factors depending on the technology used and related factors such as the print head/laser spot size/screen resolution, build orientation, materials, geometric features, and their topology. Other factors that affect dimensional accuracy include the precision of the linear stage positioning, post-treatment procedures, particle size, and layer thickness [1–3].

Regarding each of the above manufacturing technologies, the dimensional accuracy of a component part can be evaluated through its size and shape by changing the printing parameters and/or fabrication processes. While the underlying technology used in 3D printing methods have largely remained the same in recent years, nevertheless, the recent technological advancements have led to the next generation of 3D printers, which are smaller, relatively inexpensive, and more efficient compared to the traditional SLA techniques [4].

## Fabrication process

The entire fabrication process of SLA technique involves three different phases. The preparatory phase involves the use of a CAD or slicing software to set the build orientation which generates the support structure and ‘slice’ or ‘layer’ of the model and supports. The actual build is constructed in the second phase, and the third phase involves post-curing the fabricated structure, removing of the support structures, and then finishing and polishing the finished product [5]. In these phases, the build parameters are commonly interrelated and have been reported to have a significant influence on the surface quality, mechanical properties, and dimensional accuracy of the complete fabricated object [6]. Additionally, the build time and the time required for finishing of the printed object are also dependent on the selected build parameters [2]. For example, if the build has thinner layer heights, then the total print time will increase as the object will be divided into more layers.

A study by Alharbi et al. examines the effect of build angle and support configuration (thick versus thin support) on the dimensional accuracy of 3D printed full-coverage dental restorations [7]. In this study, the results suggest that the build angle and support structure configuration have a significant influence on the dimensional accuracy of 3D printed restorations. The study concludes that a build angle of 45°–90° should offer the model print the highest dimensional accuracy and self-supported geometry. As a result, this allows for the smallest necessary support surface area and reduces the time needed for finishing and polishing. However, a trade-off is created as the increased angulation may increase the total model height, and therefore, it will increase print time [7].

In another study that examined whether build direction had an effect on the mechanical properties, it was reported that materials printed vertically have higher compressive strength than those printed horizontally [8]. A more recent study found that the build angle or layer orientation influences the flexural strength of the hybrid resin material printed using the SLA technique. The study discusses that vertically printed specimens had a statistically significantly lower mean flexure strength of 88.2 MPa compared to 90.5 MPa of those printed horizontally [9].

## Degree of accuracy compared in different 3D printing techniques

Dental prostheses manufactured using 3D printing technologies have been shown to have an acceptable degree of accuracy and precision compared to prostheses made using conventional plaster cast models [10]. In a study by Dietrich et al., the accuracy (trueness and precision) of two different rapid prototyping techniques were compared to the physical reproduction of 3D digital orthodontist resin casts using SLA and PolyJet systems [11]. The results of this study indicate higher trueness in PolyJet replicas than in the SLA models, but the precision measurements favoured the SLA techniques. However, the study observed that both replicas have a maximum deviation of 127 μm in the dimensional errors [11], which was far below the recommended range of 300–500 μm for clinically relevant accuracy in orthodontic tests as discussed by Sweeney et al. [12]. Furthermore, the results show that polyvinyl siloxane materials provide more accurate interocclusal recordings for a successful articulation of digital models compared to other materials such as Regisil Rigid, Futar Scan, Byte Right, and Aluwax [11].

Kim et al. explored the precision and trueness of 3D printed dental models by assessing the differences in dimensions between the 3D printed models, made by fused filament fabrication (FFF), SLA, DLP, and PolyJet techniques, versus digital reference models [13]. The ‘trueness’ was defined as the proximity of a model to a true value, in which the least accurate 3D printing model produced replica casts within 260 μm of the reference models, which was still below the clinically relevant guidelines that Kim et al. were prepared to accept. The study shows that statistically significant differences existed between the various 3D printing technologies. The results found that both the PolyJet and DLP techniques had a higher precision compared to the FFF and SLA techniques, with the highest precision associated with the PolyJet technique [13].

Several other studies have also studied how both DLP and PolyJet are 3D printing technologies that provide exceptional accuracy and surface finish in dentistry [14–16]. Given that DLP and PolyJet printers are two 3D printing techniques commonly used in dentistry, Brown et al. conducted a study to assess the accuracy of using a digital model created from digital intraoral impression scanners. Various points were used to compare dimensional change including mesiodistal (crown width) and incisal/occlusal-gingival (crown height) and intercanine and intermolar widths [17]. The significance of this comparison aimed to evaluate the accuracy of the entire digital workflow. As stated in the previous studies, the findings indicate that both the DLP and PolyJet printers had clinically acceptable accuracy in the 3D printing models produced, and therefore, they can be considered as alternatives to plaster-cast storage in orthodontic practice [17].

A recent study evaluates the accuracy of 3D printed retainers compared with the conventional vacuum-formed retainers and commercially available vacuum-formed retainers [18]. The results from this study show that traditional vacuum-formed retainers have the least deviation from the original reference models (0.10–0.20 mm), followed by commercially formed retainers (0.10–0.30 mm), whereas the greatest deviation (0.10–0.40 mm) was found in 3D printed retainers [18]. However, all three workflows produce retainers that are within 0.5 mm accuracy and are therefore deemed clinically acceptable for the assessment of digital articulation [11].

### Hypothesis

The null hypothesis of the study was that no differences would be found between the various different 3D printer technologies regarding trueness and precision.

## Materials and methods

The present study involves some of the recently released 3D printers that have not yet been studied for their accuracy. Since these new printers will replace current models that may have been included in the previous studies in the literature, it is important to study whether they are statistically more or less accurate and to discuss whether these results are clinically relevant. For the purposes of this study, the use of a standardised printable object was used to measure the accuracy of these recent 3D printers.

### Test block sourcing

The test blocks were sourced from existing 3D printer units that are regularly used in dental practice by the International Digital Dental Academy committee and board. Some test blocks were also sourced from manufacturers who complied with the data collection methods below. Other than the data collection method, no specific information was given regarding the actual virtual block size to rule out user bias. Where blocks could only be sourced individually, other sources were found to give a more rounded and less biased production.

### 3D printers in the study

The printers used in the present in vitro study are summarised in Table 1.

**Table 1:** The 3D printers used in this study.

Name	Technology	Build platform size	Max print speed	Specification
Anycubic Photon, Shenzhen, China	LCD-based SLA 3D Printer (wavelength 405 nm)	115 × 65 × 155 mm	20 mm/h	XY DPI: 47 μm (2560*1440)
Anycubic Photon S, Shenzhen, China	LCD-based SLA 3D Printer (wavelength 405 nm)	115 × 65 × 165 mm	20 mm/h	XY DPI: 47 μm (2560*1440)
Asiga MAX UV, Sydney, Australia	DLP (UV LED 385 or 405 nm)	119 × 67 × 75 mm	20 mm/h	XY DPI: 62 μm (1920*1080)
Elegoo Mars, Shenzhen, China	LCD-based SLA 3D Printer (wavelength 405 nm)	119 × 68 × 155 mm	22.5 mm/h	XY DPI: 47 μm (2560*1440)
Envisiontec Vida HD, Envisiontec Inc, USA	DLP (UV LED 385 nm) cDLM	140 × 79 × 100 mm	47 mm/h	XY DPI: 73 μm (1920*1080)
Envisiontec ONE, Envisiontec Inc, USA	DLP (UV LED 385 nm) cDLM	180 × 101 × 175 mm	45 mm/h	XY DPI: 93 μm (1920*1080)
Formlabs Form 2, Formlabs, USA	SLA (UV laser 405 nm)	350 × 330 × 520 mm	20 mm/h	Laser spot 140 μm
Formlabs Form 3, Formlabs, USA	SLA (UV laser 405 nm)	145 × 145 × 185 mm	30 mm/h	Laser spot 85 μm
Nextdent 5100, 3D Systems, Netherlands	DLP (UV LED 405 nm)	124.8 × 70.2 × 196 mm	65 mm/h	XY DPI: 65 μm (1920*1080)
Creo, Planmeca, Finland	DLP (UV LED 405 nm)	130 × 81.5 × 130 mm	10 mm/h	XY DPI: 68 μm (1920*1080)
Sol, Ackuretta, Taiwan	LCD-based SLA 3D Printer (wavelength 385 or 405 nm)	128 × 80 × 120 mm	45 mm/h	XY DPI: 49 μm (2560*1440)
D4K Pro, Envisiontec Inc, USA	DLP (UV LED 385 nm) cDLM	148 × 83 × 110 mm	45 mm/h	XY DPI: 50 μm Enhanced Resolution: 25 μm (1920*1080)

### Design of the study

#### Data collection method

All test blocks were printed using the same settings with 100 micron Z layer thickness and the print time set to standard where applicable.

Post print processing and treatments were conducted in accordance with the manufacturers’ instructions, and the workflows included an alcohol wash and curing appropriately instructed for that resin.

All print test blocks were printed using the same positioning, in other words they were centralised on the print build platform. No supports were used to print each model and all prints were printed using the manufacturers software with all software being the latest available version as of April 2021.

**Fig. 1:**
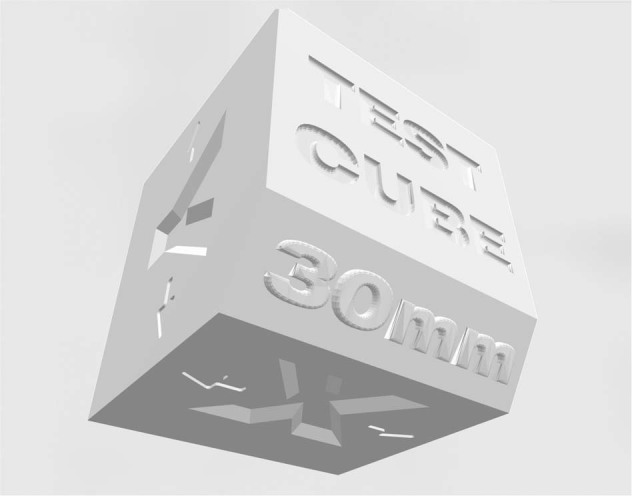
The test cube STL. The 3D STL is shown virtually which was printed on each printer in the study.

For the purposes of this study, the design of model as an openly available cuboid 3D Printing calibration model (Fig. 1) was chosen to allow the study of the distortion upon printing in each of the *XYZ* axes. As the cube is a precise shape, variances in technology will have an impact upon these printed models in terms of a precise border being accurately printed in the *XY* depending on LED size, laser spot size etc. The accuracy of the *Z* axis movements will impact the Z measurements and thus accuracy in that dimension.

It is important to study this precise shape in terms of XYZ measurements as model distortion can be harder to ascertain in which dimension the distortion occurs if a tooth or arch model is used and overall comparative scans can be deceptive in terms of an analysis of a singular dimensional distortion.

Another benefit of a pre-existing precise STL is that this master STL can be easily compared once scanned using CloudCompare measurement and 3D comparison tool.

All printers were calibrated prior to use according to the manufacturers’ recommendations and instructions.

The resins used were the manufacturers’ own dental model resins and were mixed or rolled before printing according to standard recommendations and procedures.

The layer height of 100 microns was used as this is a common layer height used for dental parts. For example, in a study by Alshamrani et al. [19] the layer height of 100 microns is recommended as this produced a higher flexural strength than a smaller layer height.

Hundred microns refers only to the vertical layer height ie the *Z* axis and does not refer to the XY dimensional accuracy. As all prints should be printing the same number of 100 micron layers to reproduce the master STL, this layer height should have no impact on the measurement of the accuracy in the Z dimension between printers.

### Measurement

To measure the resulting blocks a digital scan was taken using a Dentsply Sirona Ineos X5 lab scanner of each block. To measure the XYZ dimensions of each block produced on each printer, the captured STLs were compared using CloudCompare and the deviation compared to the Master STL recorded in each dimension.

The STL model of each print was acquired with each of the above printers. This structured light lab scanner is accredited to be accurate within 2.1 microns (ISO 12836) [20] A sample size of 10 for each printer was determined by using a sample size calculation with 95% confidence level and a margin of error of 5%. This has been confirmed by several authors to be acceptable to obtain statistically significant results [21–23].

Each measurement was taken from the central axis of that dimension.

### 3D deviation

**Fig. 2:**
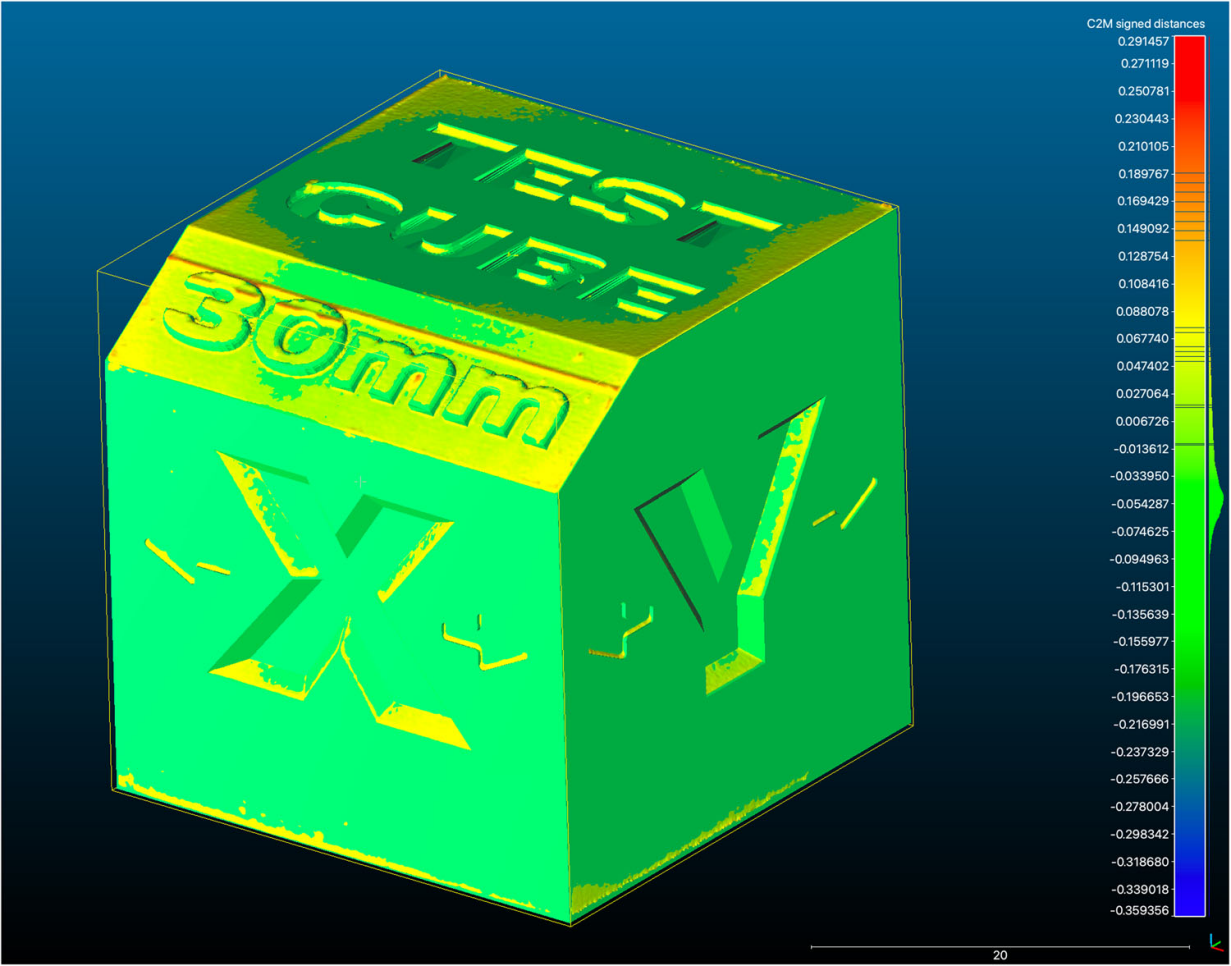
CloudCompare colour map. An example of the scanned test object overlaid with Master STL which was performed for each measurement of each print.

The CloudCompare software allows the generation of a colorimetric map of the deviation across the surface of the STL mesh as compared to the master STL, quantified at specific points allowing an overall 3D deviation comparison or a point to point analysis (Fig. 2). The colour map indicates deviation inward (blue) or outward (red), while green indicates minimal deviation. The same C2M colour deviation scale was employed to illustrate the minimum and maximum deviations for each comparison. The colour scale ranged from a maximum and minimum deviation of +200 (outward/red) and −200 μm (inward/blue). The software allows measurement deviation. across planes of XYZ.

### Main Method

The method of the study involves the use of a standardised test block that is 30 mm by 30 mm exactly. This test block will then be printed on each printer several times based on the sample size calculation then using the data collection method above;

Assessing the deviation of the X (horizontal) dimension of the printed test cube compared to the planned virtual test object.

Assessing the deviation of the Y (vertical) dimension of the printed test cube compared to the planned virtual test object.

Assessing the deviation of the Z (lateral) dimension of the printed test cube compared to the planned virtual test object.

### Evaluating trueness

For trueness, the master model scans using the Ineos X5 were used as a baseline measurement against each printed Model. Each of the ten aligned and cut scans from each of the printers in the in vitro study was brought into CloudCompare (an open 3D point cloud and mesh processing and comparison software), where the scans were further aligned and calibrated using the fine alignment algorithm. Each data set was then compared with the master STL using the CloudCompare 3D analysis best-fit algorithm. Trueness was defined as the mean deviation value for each of the XYZ dimensions. The results were recorded along with the standard deviation for each.

### Evaluating precision

All possible pairwise comparisons were made using a one-way analysis of variance for independent groups, with a Tukey significance level of 0.05, of multiple comparisons using SPSS 26 by IBM [24]. Bartlett’s test was used to test the homogeneity of variances. Precision was defined from the superimposition between the different scans made with the same intraoral scanner. The comparisons available for each printer were calculated and the precision of each printer was then expressed as a mean.

## Results

**Fig. 3:**
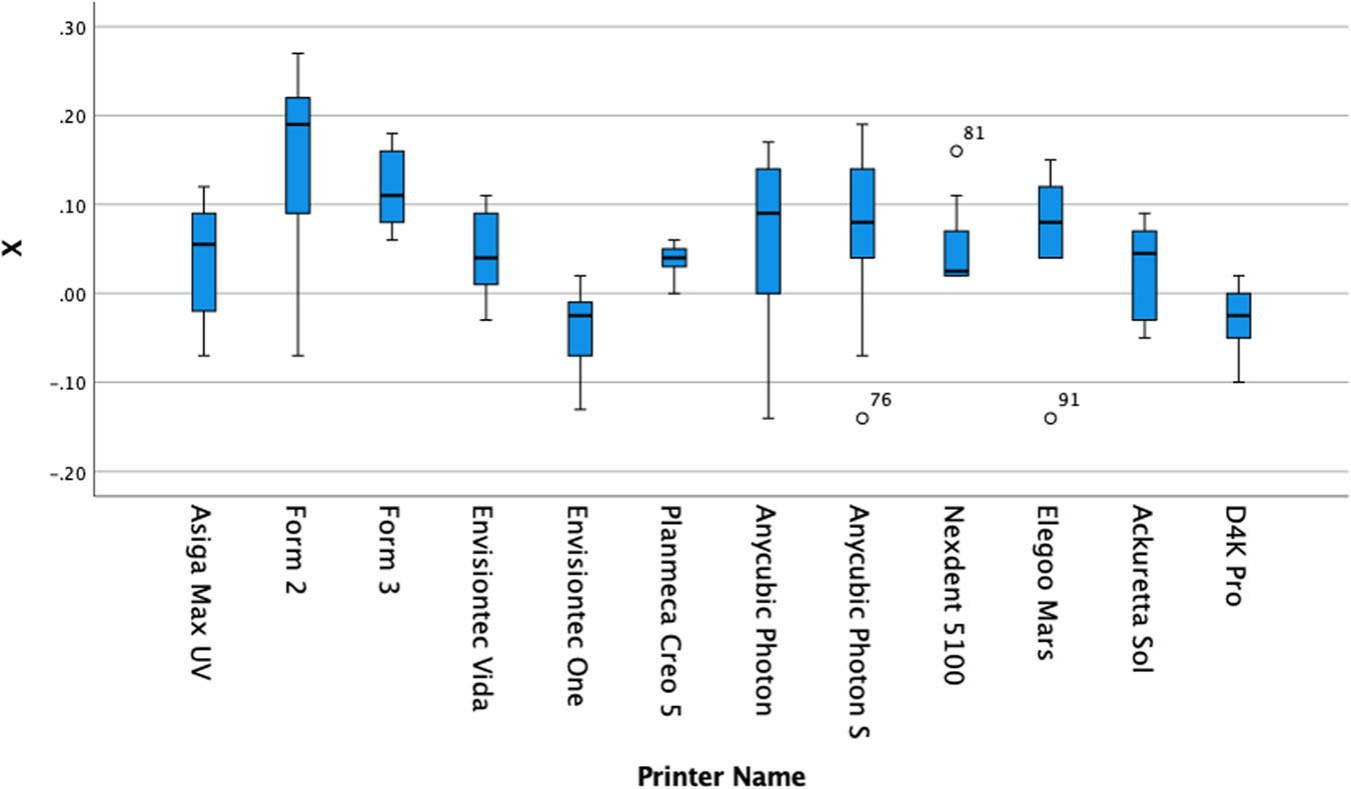
Box plot of X data set for each printer in the present study. The distribution of numerical data of the measurements of the X dimension for each print and skewness through displaying the data quartiles and averages. The y-axis show the numerical data in terms of deviation from the actual size in microns and the x-axis labels each data set in terms of which printer it applies to.

**Fig. 4:**
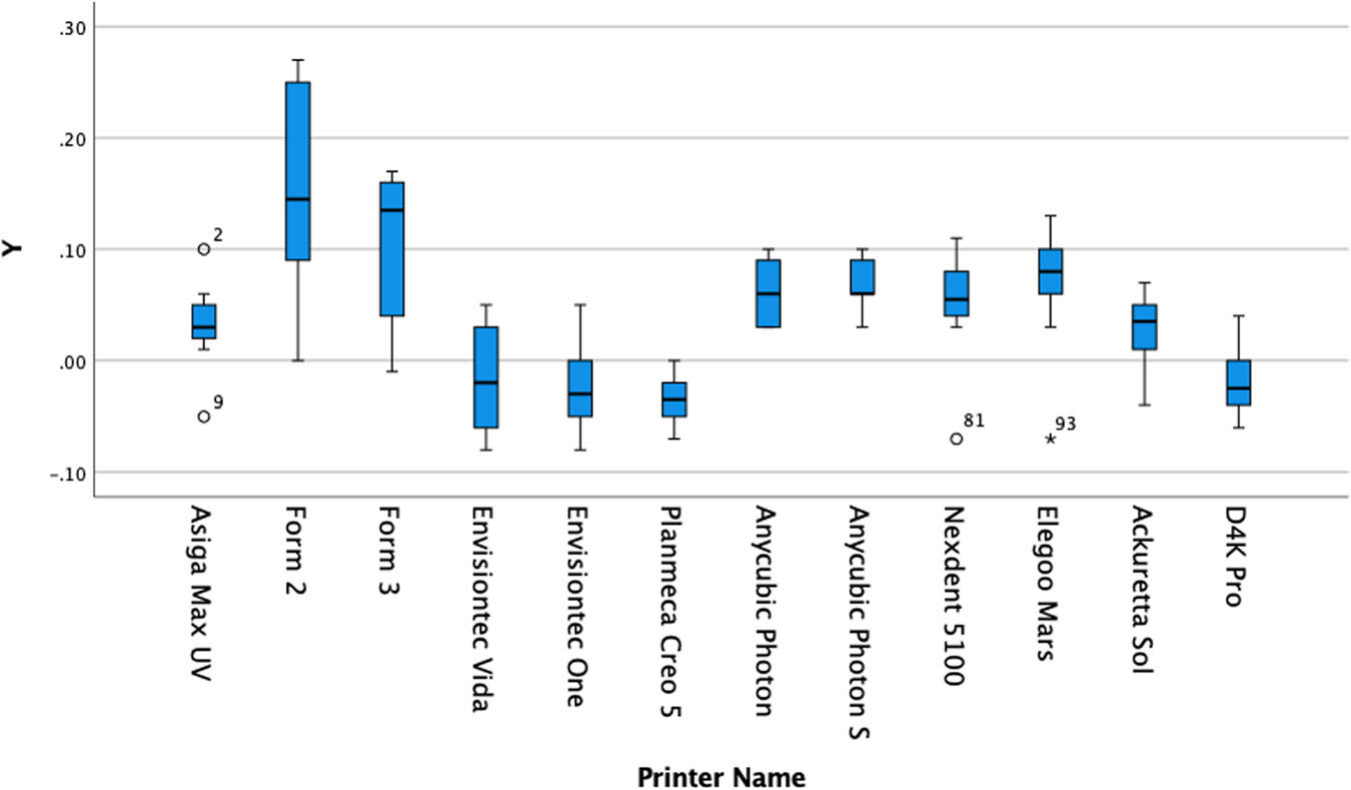
Box plot of Y data set for each printer in the present study. The distribution of numerical data of the measurements of the Y dimension for each print and skewness through displaying the data quartiles and averages. The y-axis show the numerical data in terms of deviation from the actual size in microns and the x-axis labels each data set in terms of which printer it applies to.

**Fig. 5:**
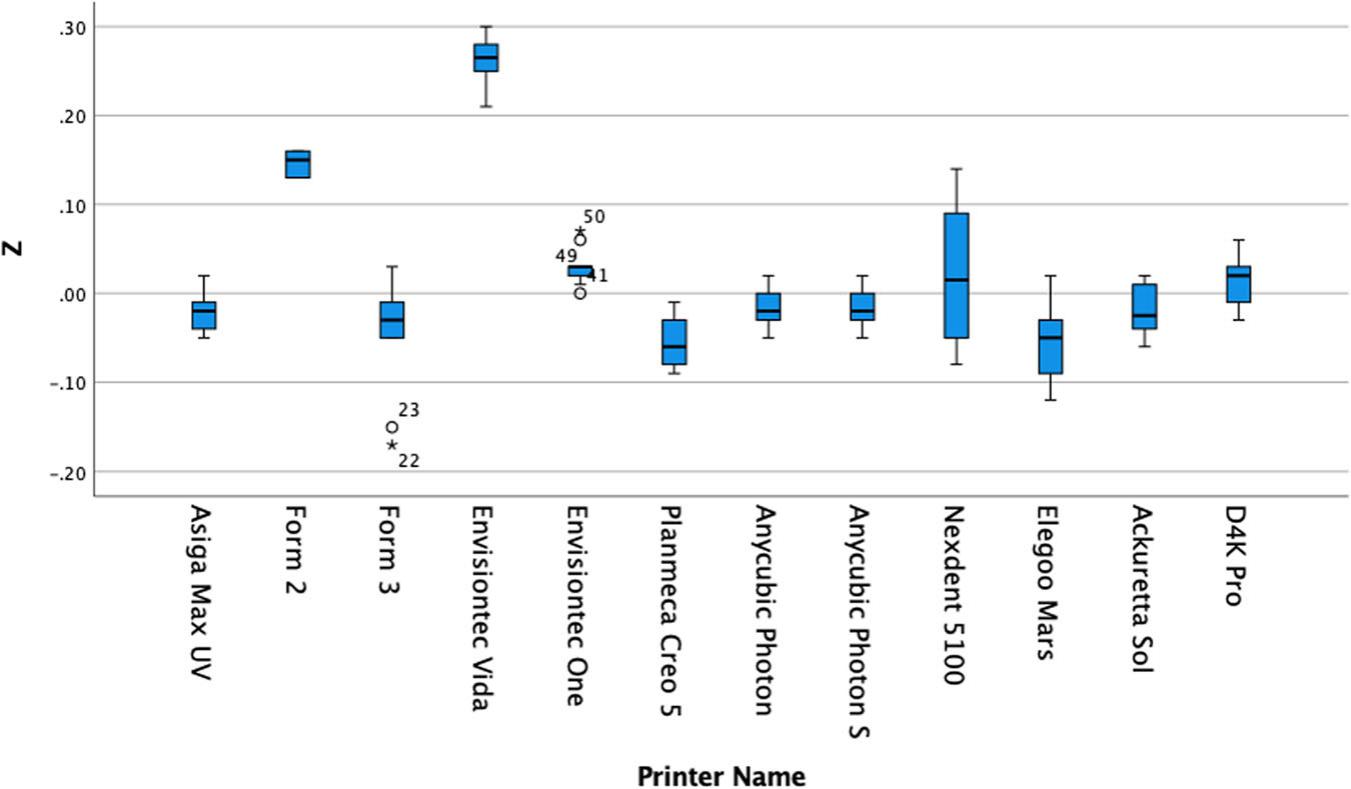
Box plot of Z data set for each printer in the present study. The distribution of numerical data of the measurements of the Z dimension for each print and skewness through displaying the data quartiles and averages. The y-axis show the numerical data in terms of deviation from the actual size in microns and the x-axis labels each data set in terms of which printer it applies to.

The results are summarised in Tables 2–5 and in Figs. 3–5.

**Table 2:** Mean deviation of each printer in comparison to the master STL.

Name	*X* axis error mean (±SD)	*Y* axis error mean (±SD)	*Z* axis error mean (±SD)
Asiga Max UV	0.041 (±0.064)	0.032 (±0.038)	−0.021 (±0.020)
Form 2	0.142 (±0.111)	0.149 (±0.094)	0.146* (±0.012)
Form 3	0.116* (±0.042)	0.108 (±0.067)	−0.047 (±0.064)
Envisiontech Vida	0.045 (±0.047)	−0.019 (±0.045)	0.262* (±0.026)
Envisiontech One	−0.035 (±0.045)	−0.028 (±0.037)	0.030 (±0.021)
Planmeca Creos	0.038* (±0.016)	−0.036 (±0.022)	−0.053* (±0.027)
Anycubic Photon	0.064 (±0.103)	0.061* (±0.026)	−0.016 (±0.025)
Anycubic Photon S	0.064 (±0.101)	0.068* (±0.026)	−0.016 (±0.025)
Nexdent 5100	0.053 (±0.048)	0.051 (±0.049)	0.019 (±0.079)
Elegoo Mars	0.072 (±0.083)	0.056 (±0.051)	−0.044 (±0.035)
Ackuretta Sol	0.030 (±0.052)	0.025 (±0.034)	0.023 (±0.028)
D4K Pro	−0.028 (±0.037)	0.021 (±0.030)	0.014 (±0.027)
Significant	≤0.05 (C.I. 95%)	≤0.05 (C.I. 95%)	≤0.05 (C.I. 95%)

**Table 3:** Tukey homogenous subsets of compared means for the X measurement (subset for alpha = 0.05).

*X* axis	Name	*N*	1	2	3
Tukey HSD^a^	Envisiontech One	10	−0.0350		
	D4K Pro	10	−0.0280		
	Ackuretta Sol	10	0.0300	0.0300	
	Planmeca Creos	10	0.0380	0.0380	
	Asiga Max UV	10	0.0410	0.0410	0.0410
	Envisiontech Vida	10	0.0450	0.0450	0.0450
	Nexdent 5100	10	0.0530	0.0530	0.0530
	Anycubic Photon S	10	0.0640	0.0640	0.0640
	Anycubic Photon	10	0.0640	0.0640	0.0640
	Elegoo Mars	10	0.0650	0.0650	0.0650
	Form 3	10		0.1160	0.1160
	Form 2	10			0.1420
	Sig.		0.065	0.198	0.059
Tukey 8^2^	Envisiontech One	10	−0.0350		
	D4K Pro	10	−0.0280		
	Ackuretta Sol	10	0.0300	0.0300	
	Planmeca Creos	10	0.0380	0.0380	
	Asiga Max UV	10	0.0410	0.0410	0.0410
	Envisiontech Vida	10	0.0450	0.0450	0.0450
	Nexdent 5100	10	0.0530	0.0530	0.0530
	Anycubic Photon S	10	0.0640	0.0640	0.0640
	Anycubic Photon	10	0.0640	0.0640	0.0640
	Elegoo Mars	10	0.0650	0.0650	0.0650
	Form 3	10		0.1160	0.1160
	Form 2	10			0.1420

Means for groups in homogeneous subsets are displayed. ^a^Uses Harmonic mean sample size = 10.000; subset for alpha = 0.05.

**Table 4:** Tukey homogenous subsets of compared means for the Y measurement (subset for alpha = 0.05).

*Y* axis	Name	*N*	1	2	3		
Tukey HSD^a^	Planmeca Creos	10	−0.0360				
	Envisiontech One	10	−0.0280				
	D4K Pro	10	−0.0210				
	Envisiontech Vida	10	−0.0190	−0.0190			
	Ackuretta Sol	10	0.0250	0.0250	0.0250		
	Asiga Max UV	10	0.0320	0.0320	0.0320		
	Nexdent 5100	10		0.0510	0.0510	0.0510	
	Anycubic Photon	10			0.0610	0.0610	
	Elegoo Mars	10			0.0660	0.0660	
	Anycubic Photon S	10			0.0680	0.0680	
	Form 3	10				0.1080	0.1080
	Form 2	10					0.1490
	Sig.		0.082	0.064	0.695	0.268	0.754
Tukey 8^2^	Planmeca Creos	10	−0.0360				
	Envisiontech One	10	−0.0280				
	D4K Pro	10	−0.0210				
	Envisiontech Vida	10	−0.0190	−0.0190			
	Ackuretta Sol	10	0.0250	0.0250	0.0250		
	Asiga Max UV	10	0.0320	0.0320	0.0320		
	Nexdent 5100	10		0.0510	0.0510	0.0510	
	Anycubic Photon	10			0.0610	0.0610	
	Elegoo Mars	10			0.0660	0.0660	
	Anycubic Photon S	10			0.0680	0.0680	
	Form 3	10				0.1080	0.1080
	Form 2	10					0.1490

Means for groups in homogeneous subsets are displayed. ^a^Uses harmonic mean sample size = 10.000; subset for alpha = 0.05.

**Table 5:** Tukey homogenous subsets of compared means for the Z measurement (subset for alpha = 0.05).

*Z* axis	Name	*N*	1	2	3	
Tukey HSD^a^	Elegoo Mars	10	−0.0540			
	Planmeca Creos	10	−0.0530			
	Form 3	10	−0.0470			
	Ackuretta Sol	10	−0.0230	−0.0230		
	Asiga Max UV	10	−0.210	−0.210		
	Anycubic Photon	10	−0.0160	−0.0160		
	Anycubic Photon S	10	−0.0160	−0.0160		
	D4K Pro	10		0.0140		
	Nexdent 5100	10		0.0190		
	Envisiontech One	10		0.0300		
	Form 2	10			0.1460	
	Envisiontech Vida	10				0.2620
	Sig.		0.537	0.096	1.000	1.000
Tukey 8^2^	Elegoo Mars	10	−0.0540			
	Planmeca Creos	10	−0.0530			
	Form 3	10	−0.0470			
	Ackuretta Sol	10	−0.0230	−0.0230		
	Asiga Max UV	10	-0.210	−0.210		
	Anycubic Photon	10	−0.0160	−0.0160		
	Anycubic Photon S	10	−0.0160	−0.0160		
	D4K Pro	10		0.0140		
	Nexdent 5100	10		0.0190		
	Envisiontech One	10		0.0300		
	Form 2	10			0.1460	
	Envisiontech Vida	10				0.2620

Means for groups in homogeneous subsets are displayed. ^a^Uses harmonic mean sample size = 10.000; subset for alpha = 0.05.

A wide variation in the results exists in the present study, and the printers could be grouped according to their consistent accuracy.

When grouped into homogenous subsets, the cheapest 3D printers in the group, namely the Anycubic printers and the Elegoo Mars, are statistically not dissimilar to the higher priced Envisiontec and Asiga Max UV or even the mid-priced Ackuretta and Formlabs printers in the X and Z dimensions. However, the Envisiontec One and D4K Pro, Ackuretta Sol and Asiga Max UV were statistically superior in terms of precision with a consistently accurate Y dimension.

Although these printers use different technologies to print, no specific type of printer technology is more accurate than the others.

## Discussion

The null hypothesis was proved to be true, in that no significant differences were found among the various technologies of 3D printing regarding trueness and precision. The evolution of 3D printers that leads to budget printers being as statistically accurate as expensive printers is remarkable. Whilst clear differences in the mean error between the printers were found, the performance of these budget class printers is considered exceptional.

In terms of each dimension, the lowest three mean errors recorded were found to be:

X Dimension;

The Envsiontec D4K Pro (−0.028 ± 0.037), the Ackuretta Sol (0.030 ± 0.052), the Envisiontec One (−0.035 ± 0.045).

Y Dimension;

The Envsiontec D4K Pro (−0.021 ± 0.030), the Ackuretta Sol (0.025 ± 0.034), the Envisiontec One (−0.028 ± 0.037).

Z Dimension;

The Envsiontec D4K Pro (−0.014 ± 0.027), the Anycubic Photon (S) (−0.016 ± 0.025), the Asiga Max UV (−0.021 ± 0.020).

Whilst the 3D printers specific to the Dental market have clear advantages in terms of speed and availability of open and calibrated resin settings, the statistical superiority of these printers in terms of dimensional accuracy is not proven. Therefore general dental practices with a lower budget can also access 3D Printing technologies to benefit patients with an easier way of creating a model in a more predictable and repeatable way to alleviate problems or complications encountered in conventional methods when making impressions [25].

The purpose of this study is to address the issues regarding precision/trueness and accuracy by comparing different printer technologies. This study assessed these parameters for 12 3D printers using four different technologies to print resin models chairside. In terms of the four various 3d printing technologies, namely SLA laser, DLP, DLP cDLM and LCD-based SLA, the results show there is no technology statistically superior to the others. Even in the Y Dimension, where there were no budget printers within the most accurate homogenous subset (consisting of the Planmeca Area 5, Envisiontec One, D4K Pro, Envisiontec Vida HD, Ackuretta Sol and Asiga Max UV) the printers within that subset used different technologies.

This study is the most up to date research on the most recent printers that have been released as of the end of 2021. In the present study, only one clinician performed the measurements on the models to produce the data set for each printer, and each measurement was taken on a recently calibrated printer and within the same time frame after the same postprocessing. A sole clinician performing these measurements is important as variation in either of these can affect the accuracy of the model in terms of contraction and final shape [26–28].

The fast-paced changes and developments in modern dentistry within CAD/CAM, digital impression registration, and chairside production along with the quick development of new software options mean that 3D printing will most likely be more frequently used within dentistry as the technology develops further. Moreover, 3D printing is likely to quickly become an even greater factor in developing modern dentistry. Whilst an abundance of data indicates that quick and accurate data capturing of the intraoral environment is possible, there is a paucity of data relating to the conversion of this data to the 3D printed model, especially with newer machines [29–32].

Whilst there are no statistical differences in terms of X and Z accuracy, there are advantages to 3D printers that are developed specifically to be used by dental surgeons. In comparing the technology of the printers within this study with the highest mean accuracy recorded there are also some features which may present advantages to certain dental clinics and for a variety of specific resin needs. The Asiga Max UV has in-built technology such as pressure sensors in the DLP display to increase speed by detection of separation. One of the printers in this study, the Ackuretta Sol, is an entirely dental specific 3D printer and has a wide range of resin profiles that are calibrated and in built into the slicing software. Some manufacturers have also differentiated themselves from budget printers by developing resins which are licensed exclusively for their brand. This is the case with the D4K Pro by Envisontec which the results showed had the highest overall mean accuracy. The D4K Pro, along with other Envisontec branded printers, have biomedical resins licenced exclusively for their platform, for example the Flexcera resin used for digital dentures and restorations.

This study on the latest 3D printers shows that they can produce results that are accurate to within 30 microns in each of the XYZ dimensions. For the errors of the printers in the present study, the overall combined error should be within a clinically acceptable level of under 100 microns. These printers exceeded expectations and they are all worthwhile to use in clinical practice.

## Conclusions

This study shows that the current range of 3D printers can produce clinically acceptable levels of accuracy. The present study also shows that there is no statistical difference in the results of budget printers and more expensive printers. This study confirms that all 12 of the 3D printers can produce a reliable, reproducible model. However, the printing of dental arches and restorations is more challenging in terms of complex and fine details and this deserves further investigation. Following this study, further research is needed on these printers in various settings, and the evidence of their accuracy and strength of materials must be confirmed in a clinical setting.

## References

[1] Dimitrov D, Van Wijck W, Schreve K, De Beer N (2006). Investigating the achievable accuracy of three dimensional printing. Rapid Prototyp J.

[2] Puebla K, Arcaute K, Quintana R, Wicker RB (2012). Effects of environmental conditions, aging, and build orientations on the mechanical properties of ASTM type I specimens manufactured via stereolithography. Rapid Prototyp J.

[3] Favero CS, English JD, Cozad BE, Wirthlin JO, Short MM, Kasper FK (2017). Effect of print layer height and printer type on the accuracy of 3-dimensional printed orthodontic models. Am J Orthod Dentofac Orthop.

[4] Al-Imam H, Gram M, Benetti AR, Gotfredsen K (2018). Accuracy of stereolithography additive casts used in a digital workflow. J Prosthet Dent.

[5] Tapie L, Lebon N, Mawussi B, Fron-Chabouis H, Duret F, Attal JP (2015). Understanding dental CAD/CAM for restorations- accuracy from a mechanical engineering viewpoint. Int J Comput Dent.

[6] Lebon N, Tapie L, Duret F, Attal JP (2016). Understanding dental CAD/CAM for restorations- dental milling machines from a mechanical engineering viewpoint. Part A: chairside milling machines. Int J Comput Dent.

[7] Alharbi N, Osman RB, Wismeijer D (2016). Factors influencing the dimensional accuracy of 3D-printed full-coverage dental restorations using stereolithography technology. Int J Prosthodont.

[8] Alharbi N, Osman RB, Wismeijer D (2016). Effects of build direction on the mechanical properties of 3D-printed complete coverage interim dental restorations. J Prosthet Dent.

[9] Alharbi N, van de Veen AJ, Wismeijer D, Osman RB (2019). Build angle and its influence on the flexure strength of stereolithography printed hybrid resin material. An in vitro study and a fractographic analysis. Mater Technol.

[10] Han T, Kundu S, Nag A, Xu Y (2019). 3D printed sensors for biomedical applications: a review. Sensors.

[11] Dietrich CA, Ender A, Baumgartner S, Mehl A (2017). A validation study of reconstructed rapid prototyping models produced by two technologies. Angle Orthod.

[12] Sweeney S, Smith DK, Messersmith M (2015). Comparison of 5 types of interocclusal recording materials on the accuracy of articulation of digital models. Am J Orthod Dentofac Orthop.

[13] Kim SY, Shin YS, Jung HD, Hwang CJ, Baik HS, Cha JY (2018). Precision and trueness of dental models manufactured with different 3-dimensional printing techniques. Am J Orthod Dentofac Orthop.

[14] Katreva I, Dikova T, Abadzhiev M, Tonchev T, Dzhendov D, Simon M (2016). 3D printing in contemporary prosthodontic treatment. Scr Sci Med Dent.

[15] Oberoi G, Nitsch S, Edelmayer M, Janjić K, Müller AS, Agis H (2018). 3D printing-encompassing the facets of dentistry. Front Bioeng Biotechnol.

[16] Revilla-León M, Özcan M (2017). Additive manufacturing technologies used for 3D metal printing in dentistry. Curr Oral Health Rep.

[17] Brown GB, Currier GF, Kadioglu O, Kierl JP (2018). Accuracy of 3-dimensional printed dental models reconstructed from digital intraoral impressions. Am J Orthod Dentofac Orthop.

[18] Cole DJ, Bencharit S, Carrico CK, Arias A, Tüfekçi E (2019). Evaluation of fit for 3D-printed retainers compared with thermoform retainers. Am J Orthod Dentofac Orthop.

[19] Alshamrani AA, Raju R, Ellakwa A (2022). Effect of printing layer thickness and postprinting conditions on the flexural strength and hardness of a 3D-printed resin. Biomed Res Int.

[20] Ineos X5 Lab Scanner Accuracy Information. https://www.dentsplysirona.com/en-gb/categories/lab/cad-cam-equipment-dental-lab/ineos-x5.html#additional-information (Accessed: 01/11/2021)

[21] Nedelcu RG, Persson AS (2014). Scanning accuracy and precision in 4 intraoral scanners: an in vitro comparison based on 3-dimensional analysis. J Prosthet Dent.

[22] Mangano F, Gandolfi A, Luongo G, Logozzo S (2017). Intraoral scanners in dentistry: a review of the current literature. BMC Oral Health.

[23] Mangano FG, Hauschild U, Veronesi G, Imburgia M, Mangano C, Admakin O (2019). Trueness and precision of 5 intraoral scanners in the impressions of single and multiple implants: a comparative in vitro study. BMC Oral Health.

[24] SPSS 26 Statistics. https://developer.ibm.com/predictiveanalytics/2019/04/09/whats-new-in-spss-statistics-26/ (Accessed: 01/11/2021)

[25] Torabi K, Farjood E, Hamedani S (2015). Rapid prototyping technologies and their applications in prosthodontics, a review of literature. J Dent.

[26] Azari A, Nikzad S (2009). The evolution of rapid prototyping in dentistry: a review. Rapid Prototyp J.

[27] Hazeveld A, Huddleston Slater JJ, Ren Y (2014). Accuracy and reproducibility of dental replica models reconstructed by different rapid prototyping techniques. Am J Orthod Dentofac Orthop.

[28] Park ME, Shin SY (2018). Three-dimensional comparative study on the accuracy and reproducibility of dental casts fabricated by 3D printers. J Prosthet Dent.

[29] Revilla-León M, Gonzalez-Martín Ó, Pérez López J, Sánchez-Rubio JL, Özcan M (2017). Position accuracy of implant analogs on 3D printed polymer versus conventional dental stone casts measured using a coordinate measuring machine. J Prosthodont.

[30] Revilla-León M, Meyers MJ, Zandinejad A, Özcan M (2019). A review on chemical composition, mechanical properties, and manufacturing work flow of additively manufactured current polymers for interim dental restorations. J Esthet Restor Dent.

[31] Richert R, Goujat A, Venet L, et al. Intraoral scanner technologies: a review to make a successful impression. J Healthc Eng. 2017;2017:8427595. 10.1155/2017/8427595. 10.1155/2017/8427595 PMC5605789 29065652

[32] Rovelo P. Additive manufacturing in the medical and dental technology industries. 2019. https://3dprintingindustry.com/news/additive-manufacturing-medical-dental-technology-industries-80348/.

